# Impact Wave Monitoring in Soil Using a Dynamic Fiber Sensor Based on Stimulated Brillouin Scattering

**DOI:** 10.3390/s150408163

**Published:** 2015-04-08

**Authors:** Qingsong Cui, Sibel Pamukcu, Mesut Pervizpour

**Affiliations:** 1 National Key Laboratory of Science and Technology on Tunable Laser, Harbin Institute of Technology, Harbin 150001, China; 2 Department of Civil and Environmental Engineering, Lehigh University, Bethlehem, PA 18015, USA; E-Mails: sp01@lehigh.edu (S.P.); mepa@lehigh.edu (M.P.)

**Keywords:** Brillouin scattering, underground, infrastructure, dynamic strains, vibration

## Abstract

The impact wave response of soil due to a ball drop is monitored on a 30.5 cm by 30.5 cm square soil box using a fiber sensor with dynamic strain sensing capability. The experiments are conducted in real time using a simple one-laser one-modulator configuration with stimulated Brillouin scattering. The embedded BOTDA sensor grid successfully monitored the distribution and evolution of the inner strains of a sand bed during a mass impact on its surface. The measurement of the distributed dynamic strains was possible in several milliseconds and with 1 cm actual location resolution. This paper presents a time-domain signal analysis utilized for determining the dynamic strains in embedded fiber sensor. The results demonstrate the method to be a promising one for detection of subsurface vibration and movement in geotechnical Structure Health Monitoring (SHM).

## 1. Introduction

The availability of various sensing techniques has become increasingly important in health monitoring of the underground infrastructure and the environment [1,2,3,4,5,6]. Typically, the desired sensing system is one that is easily embedded into the structure, easy to operate, reliable and accurate for long-term continuous operation and that is sensitive to preemptive warning. The sensors used to capture sightless geo-events in the subsurface must be buried at a certain depth. Most of these sensors are targeted at monitoring multiple indices including moisture, temperature, pressure, vibration or other physical variables. Some remote subsurface sensors have also been designed and employed in military and security applications to monitor ground impact or site intrusions. Among the commonly used sensors, wired ones are often visible and can be prone to failure due to extreme environmental factors, which makes these sensors unsuitable for security and distributed applications. Issues such as power consumption, battery life and maintaining the transmission strength remain unresolved in most field applications of wireless sensors [2]. Optical fiber sensors have several advantages such as small size, light weight, immunity to electromagnetic interference (EMI), high temperature possibility, wide bandwidth, high sensitivity, and distributed sensing capability. Brillouin scattering is one of the distributed optical fiber sensing techniques that have been demonstrated in several studies [6,7,8,9].

The traditional pulse-based Brillouin sensors are not suitable for dynamic strain measurements because the weak Brillouin signal needs to be averaged to attain the necessary signal-noise-ratio (SNR) [10]. Ogawa [11] used Brillouin amplification to measure dynamic strain with a low spatial resolution of 100 m in a period of 2 s. This method showed signal stability problems. Hotate and Ong [12] presented a correlation-based continuous-wave technique and successfully measured the dynamic strains of a 5-cm vibrating fiber section at a sampling rate of 8.8 Hz. Pamukcu *et al.* conducted a free vibration test on simply supported steel bar measuring the oscillation frequency of 4.2 Hz which compared well to the theoretical frequency of 4 Hz [13]. Peled *et al.* presented slope assisted determination of fast distributed sensing in optical fiber, in which a specially synthesized and adaptable probe wave was used to place the Brillouin interaction on the slope of the local Brillouin gain spectrum [14]. The possibility of transfer of intensity modulation to Brillouin shift modulation has been explored by our team and others in recent years [15,16,17].

In this paper, a modulated-pulse-based reflection BOTDA sensor system is used to monitor the evolution of the internal strain response of a packed sand bed under impact load. A smart fiber grid design is envisioned to determine the impact wave response around and in the vicinity of buried infrastructure such as pipelines, piers, piles and other foundation systems. The low cost and passive features (*i.e.*, external detection and data acquisition) of a single mode fiber (SMF) make this design particularly attractive for distributed applications in the subsurface.

## 2. Measurement Principle

**Figure 1:**
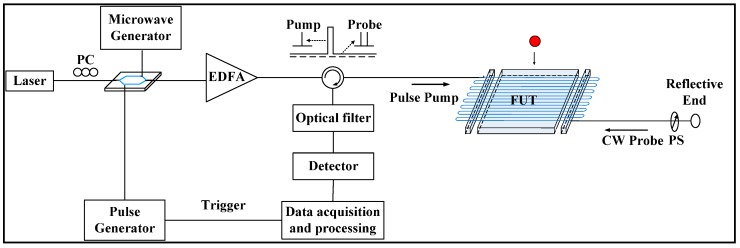
Distributed Brillouin sensor setup.

The impact wave was monitored using is a simple one-laser, one-modulator configuration BOTDA sensor system. The details of the system operation were also given in an earlier publication [16]. Figure 1 briefly describes the unique features of the dynamic sensing system. First, a combined pulse and its direct current (DC) signal are injected into the sensing fiber, where the carrier frequency is highly suppressed in the DC section. The Brillouin scattering process occurs when the reflection of the DC signal (probe wave) interacts with the pulse pump of the carrier frequency (pump wave). Sidebands modulation is used here to generate pump and probe waves simultaneously. The pulse pump is generated at high repetition (100 kHz), and the probe wave is detected at 1-GHz sampling rate. Segments storage is applied to acquire repeatedly the time domain signal generated at every sensing point, each at 100-kHz sampling rate, along the entire fiber. The sensing fiber used is SMF-28 with a pump pulse width of 6.25 ns. Finally, the intensity modulation is transferred to Brillouin shift modulation.

**Figure 2:**
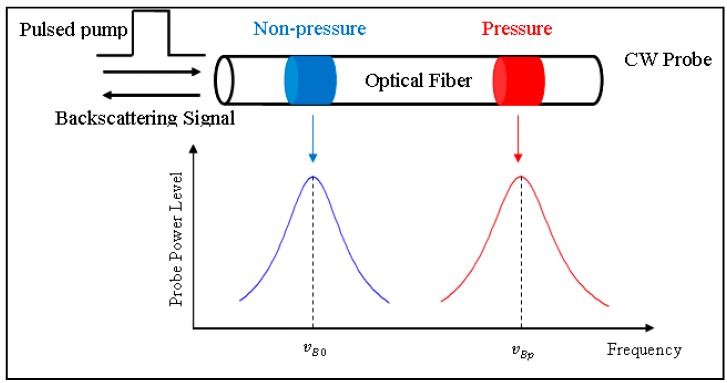
Measurement principle in BOTDA system.

Brillouin shift modulation is induced by the strain changes in the sensing fiber. The predetermined strain coefficient for the SMF-28 is 0.05941 MHz/με ≥ 16.83 με/MHz [18]. When the pulse light propagates through the optical fiber, a small fraction of light is backscattered. Part of the backscattered light is Brillouin scattering, which results from the interaction between light photons and acoustic waves (phonons). Brillouin scattering light has a frequency shift compared with the input light frequency. This shift is proportional to the acoustic velocity of the wave guide material as follows:

(1) $$v_{B}=\frac{2nV_{a}}{\text{λ}}$$

where, *n* is the refractive index, *V_a_* is the acoustic velocity, λ is the wavelength of the incident light and *ν_B_* is the Brillouin shift. Temperature or strain changes will alter the acoustic velocity in the fiber, and the Brillouin shift will consequently change. In stimulated Brillouin scattering (SBS), the strain profile along the sensing fiber can be extracted by adjusting the frequency difference between the pump and probe waves, which is illustrated in Figure 2. The traveling pulse of light is scattered back from every point along the fiber. Using the velocity of light in the fiber, this time domain information is converted into location, or distance, *l*, from the detector along the fiber:

(2) l=vt/2

Here, *l* is the distance, *ν* is the light velocity in the fiber and *t* is the measurement time. The spatial resolution, *w*, is determined by the pulse width (Δτ), which is defined by the relation:

(3) w=vΔτ/2

Although the real optical spatial resolution is limited by pulse width as given Equation (3), in this demonstration the sensing fiber sections were placed parallel to each other in a grid at 1-cm intervals to achieve a better actual location resolution.

## 3. Test Configuration

**Figure 3:**
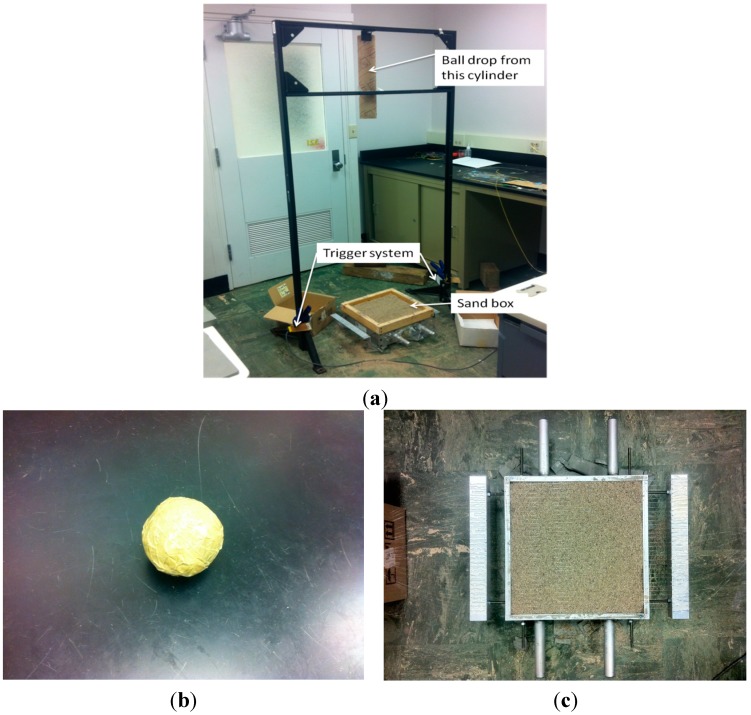
(**a**) Impact dynamic testing system; (**b**) ball; (**c**) fiber layout.

The impact wave was generated using a ball-drop test as shown in Figure 3. Figure 3a presents an overview of the guiding frame used to drop the ball from a fixed height to a target location on the sand box. The ball was guided through a cardboard cylinder attached at the top of the frame to control the line of the drop. The height of the free fall was 2 m. A reflective laser trigger system was placed close to the ball contact surface on top of the sand box. The trigger system was a compact self-contained reflective laser transmitter and a receiver with 1-ms interval of response. When the ball passed through the laser beam, the light to the receiver was blocked and, changed the output voltage. The voltage change triggered the start of data acquisition. This triggering system provided an effective approach to record the useful information during the impact interaction time with the least data size. Figure 3b shows the ball used in the experiment. Made of lightweight polymer material, the diameter of the ball was approximately 7.2-cm, weighing only 192.3 g.

Figure 3c shows the fiber placement on the surface of the 305 × 305 × 102 mm sand bed. The sand used in the experiment was Sandblast 0 (medium sand) with D10 0.5 mm and D50 1 mm. An aluminum frame was used to affix 56-cm-long strands of sensing fiber at 1-cm intervals, providing a grid of 30 fiber strands distributed along the 30-cm width of the sand bed. Parallel placement of the fiber in strands separated by 1-cm distance provided a system with the capability of 1-cm actual location resolution of measurement in the orthogonal direction. An additional layer of sand of 2-cm thickness was packed on top of this fiber grid using a wooden frame fixed on top of the aluminum box, as illustrated in Figure 3a. This arrangement enabled effectively the embedment of the fiber strands under 2-cm-thick packed sand. The fiber grid was designed so that only a pre-stretched section of the fiber worked for sensing, while the remainder of the fiber between the loops of the adjacent strands was left long and loose to assure 1-m resolution of sensing along the entire fiber.

When constructing the sensor grid assembly, one end of each strand on the grid was first epoxy glued onto the aluminum frame. Then, exactly the same amount of mass was attached to the other end of each strand to ascertain that every strand was applied the same tensile force and stretched over the same length before securing their free ends to the frame. Once each strand was glued and secured firmly on the frame the hanging masses were removed. The magnitude of the pre-strain achieved in all the strands was assumed to be uniform based on the uniform assembly protocols applied to each strand. In our photonics set up, we used a short pulse to achieve a wider dynamic range and to better discern the differences of the Brillouin shift achieved in each strand during the impact test. These adjustments and techniques were developed early through multiple pre-test trials to assure reliability of the Brillouin shift measurements.

## 4. Measurement and Results

Pre-test calibration measurements of baseline interactions of ball-sand and ball-fiber were made under static loading conditions. The fiber was scanned to acquire its initial Brillouin spectrum prior to the ball drop. This spectrum was used to isolate the dynamic strain values from the test data.

**Figure 4:**
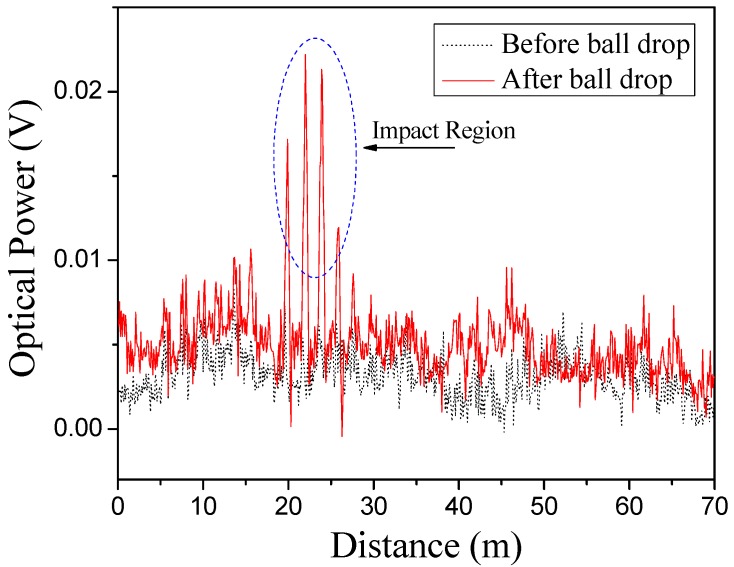
Static data along the fiber distance (11.20 GHz).

**Figure 5:**
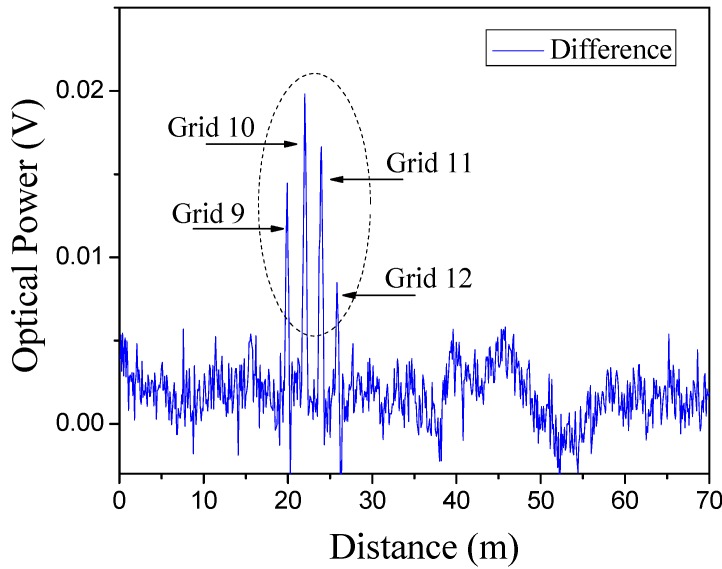
Difference of signal between pre and post ball drop (11.20 GHz).

Figure 4 shows the time domain test results before and after the ball drop. The time domain signal is based on the 11.20-GHz fixed modulation frequency and is recorded for the total length of the fiber (70 m). In Figure 4, the black colored trace (*i.e*., the dot trace) is the Brillouin intensity signal record before the ball drop. It is noted that when the modulation frequency is close to local Brillouin frequency shift, there should be 30 visible peaks, each one corresponding to one of the stretched fiber strands embedded in the sand. It is hard to discern the total of 30 peaks in Figure 4 because the modulation frequency and local Brillouin frequency shift are mismatching for most as the Brillouin gain signal is low at 11.20 GHz. The red colored trace (*i.e*., the solid trace) is the Brillouin intensity signal record after the ball drop. In general, the two traces are similar except for the central four peaks representing the impact region. These peaks reflect the strain caused by the ball impact in strands No. 9, 10, 11 and 12, owing to the increase in intensity of Brillouin shift matching. Figure 5 clearly shows the impacted and un-impacted regions, where the difference between the before and after ball drop time domain signals is plotted.

**Figure 6:**
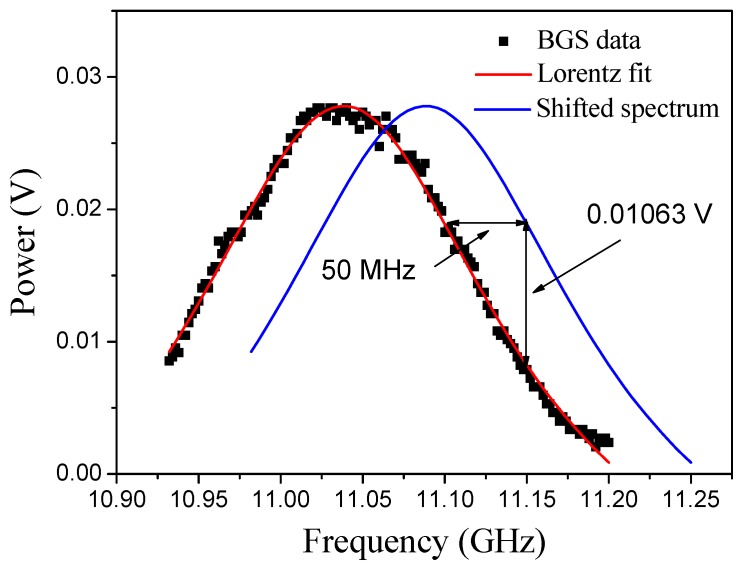
Static spectra before and after the ball drop for Grid 9.

In fast dynamic testing, it is not possible to capture the Brillouin spectrum in all the strands in the allotted time of the event. Furthermore, the Brillouin intensity is different for every peak (*i.e.*, position along fiber); therefore, it is necessary to convert the Brillouin intensity signal from the time domain to frequency domain. To illustrate, static Brillouin spectra, fitted with Lorentz curves, are shown for Grid 9 before and after the ball-drop in Figure 6. The Brillouin shift difference and the corresponding power difference are determined as shown in the figure to obtain the linear relationship between the power amplitude and strain. In Figure 6, the square markers represent the Brillouin gain spectrum (BGS) data, and the Lorentz fit is shown as the solid line. The dotted line represents the shifted spectrum. On the graph, the power change Δ*A* is measured as 0.01063 V when the modulation frequency is fixed at 11.20 GHz. This power change corresponds to a Brillouin shift Δ*f* of 50 MHz. Therefore, the linear relationship can be expressed as:

(4) $$C_{1}=\frac{\Delta f}{\Delta A}=\frac{50}{0.01063}=4703.67\text{ MHz/V}$$

It should be noted that this relationship is satisfied when the dynamic signal shift (*i.e*., the voltage change) is on the right slope of the spectrum fit. The maximum Brillouin shift (160 MHz in this demonstration) is limited by the linear slope region of the spectrum. If the dynamic signal shift falls outside of the linear slope region, the linear transfer of shift to intensity would be inaccurate. In previous studies the following linear relation was established between the strain and Brillouin shift for the SMF-28 (Corning) fiber, also used in this experiment:

$$C_{2}=16.83\text{ με/MHz}$$

The relationship between the strain and signal amplitude can then be described as:

(5) $$C=C_{1}\times C_{2}$$

The calibration constants of optical intensity to strain computed using Equations (4) and (5) as they relate to before and after ball drop measurements of fiber grid strands No. 9, 10, 11 and 12 are given in Table 1.

**Table 1:** Relationship between amplitude and strain.

Fiber #	C_1_ (MHz/V)	C_2_ (με/MHz)	C (με/V)
9	4703.67	16.83	79,162.77
10	3974.54	16.83	66,891.51
11	5087.58	16.83	85,623.97
12	5281.18	16.83	88,882.26

**Figure 7:**
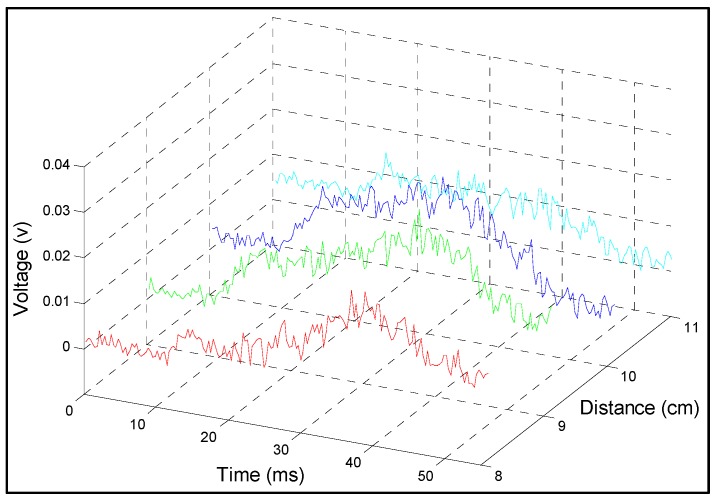
Distributed measurements of a dynamic test for the fiber strands No. 9, 10, 11, 12.

**Figure 8:**
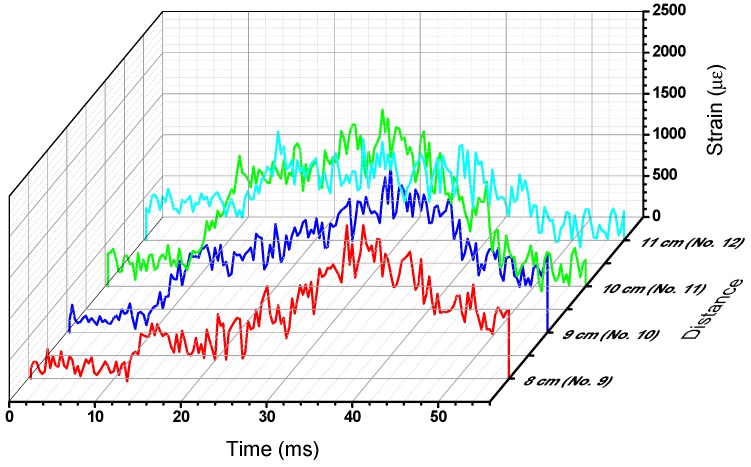
Computed strain amplitude distribution.

Calibration constant of each impacted strand were used to determine the temporal variation of the strains from the power change time domain signal obtained during the impact. The sampling rate of the sensing system was about 1 Gpts/s and the repetition rate of the pump pulse was 100 kHz/s which resulted in 100 kpts/s of sampling for each fiber strand position. Figure 7 shows the acquired temporal data for the four fiber grid strands impacted. To improve the signal noise ratio, 16 times averaging was applied in data processing. The dynamic strains were then computed using the calibration constants determined earlier (Table 1), and plotted as shown in Figure 8. Since the calibrations are linear, the temporal distribution of the strains follows closely the power distribution over time. The event is triggered at 0ms, but the impact does not take place until about 25 ms and lasts only 15 ms, when the fiber strains peak in the range 1500–2200 με.

## 5. Conclusions

A novel embeddable BOTDA sensor was used to measure dynamic subsurface strains. The effectiveness of a previously proposed sensing technique to measure dynamic strains as a result of impact loading was confirmed with ball drop experiments on a sandbox. Dynamic changes in several milliseconds and in 1 cm actual location resolution were obtained. The implications of this new measurement system is higly promising as it would enable truly distributed monitoring of vibrations and impact waves on and in vicinity of buried infrastructure where other monitoring techniques may not provide the continuous spatial and temporal coverage required.
